# C2-domain mediated nano-cluster formation increases calcium signaling efficiency

**DOI:** 10.1038/srep36028

**Published:** 2016-11-03

**Authors:** Mike Bonny, Xin Hui, Julia Schweizer, Lars Kaestner, André Zeug, Karsten Kruse, Peter Lipp

**Affiliations:** 1 Theoretical Physics, Saarland University, Saarbrücken, Germany; 2 Institute for Molecular Cell Biology, Medical Faculty, Saarland University, Homburg/Saar, Germany; 3 Cellular Neurophysiology, Center of Physiology, Hannover Medical School, Hannover, Germany

## Abstract

Conventional protein kinase Cs (cPKCs) are key signaling proteins for transducing intracellular Ca^2+^ signals into downstream phosphorylation events. However, the lifetime of individual membrane-bound activated cPKCs is an order of magnitude shorter than the average time needed for target-protein phosphorylation. Here, we employed intermolecular Förster resonance energy transfer (FRET) in living cells combined with computational analysis to study the spatial organization of cPKCs bound to the plasma membrane. We discovered Ca^2+^-dependent cPKC nano-clusters that significantly extend cPKC’s plasma-membrane residence time. These protein patterns resulted from self-assembly mediated by Ca^2+^-binding C2-domains, which are widely used for membrane-targeting of Ca^2+^-sensing proteins. We also established clustering of other unrelated C2-domain containing proteins, suggesting that nano-cluster formation is a key step for efficient cellular Ca^2+^-signaling.

Signaling through the ubiquitous second messenger Ca^2+^ requires efficient downstream transduction^1^ and relies on readout modules such as EF-hand containing, such as calmodulin, or C2-domain containing proteins, e.g. conventional protein kinase Cs (cPKCs). Activation of the ubiquitously expressed cPKCs requires binding of the second messenger Ca^2+^, plasma-membrane attachment, interaction with diacyglycerol (DAG)^2^,^3^, and results in phosphorylation of downstream target proteins^4^. However, cPKC mediated signal transduction appears inefficient, considering that the lifetimes of free and membrane-bound Ca^2+^-cPKC complexes were measured *in vitro* to be 12–15 ms and 75 ms, respectively, whereas phosphorylation of a target protein requires 150 ms^5^,^6^,^7^. Given these conditions, reliable signaling requires either to extend the lifetime of activated PKCs or decreases the time they need for phosphorylation. How live cells overcome this limitation of cPKC signaling is currently unknown.

The formation of membrane-bound homo-protein clusters is an appealing concept for explaining important features of cellular signaling. In the context of bacterial chemotaxis, large long-lived receptor clusters were shown to enhance signaling sensitivity^8^. The formation of transient extended clusters of Ras family small GTPases were associated with lipid rafts and may contribute to the discrimination between various signals by digitizing environmental stimuli^9^,^10^.

In the context of Ca^2+^-signaling evidence for the existence and contribution of nano-clusters to downstream signaling is indirect at best. *In vitro* studies reported evidence for interactions between different domains of the conventional protein kinase Cα (PKCα) that lead to dimerization^11^,^12^,^13^. These interactions were found to be important for kinase activation. In addition, the C2-domain of PKCα induces segregation of phospholipids *in vitro*^14^, which might lead to PKCα signaling complexes comprised of several PKCα molecules. Nonetheless, so far there is no evidence from studies *in vivo* for the existence, let alone for a functional role of PKCα oligomerization or nano-clustering. However, the aforementioned discrepancy between membrane residence times (<75 ms) and the time required for one phosphorylation event (>150 ms) requires the existence of some mechanism that either extends the activation lifetime of PKCα or decreases the time necessary for phosphorylation. In light of the observed roles of nano-clusters in signaling, in this work we study the existence of PKCα-nano-clusters and their possible role in signaling.

## Result and Discussion

### Membrane-bound PKCα form nano-clusters

**Figure 1:**
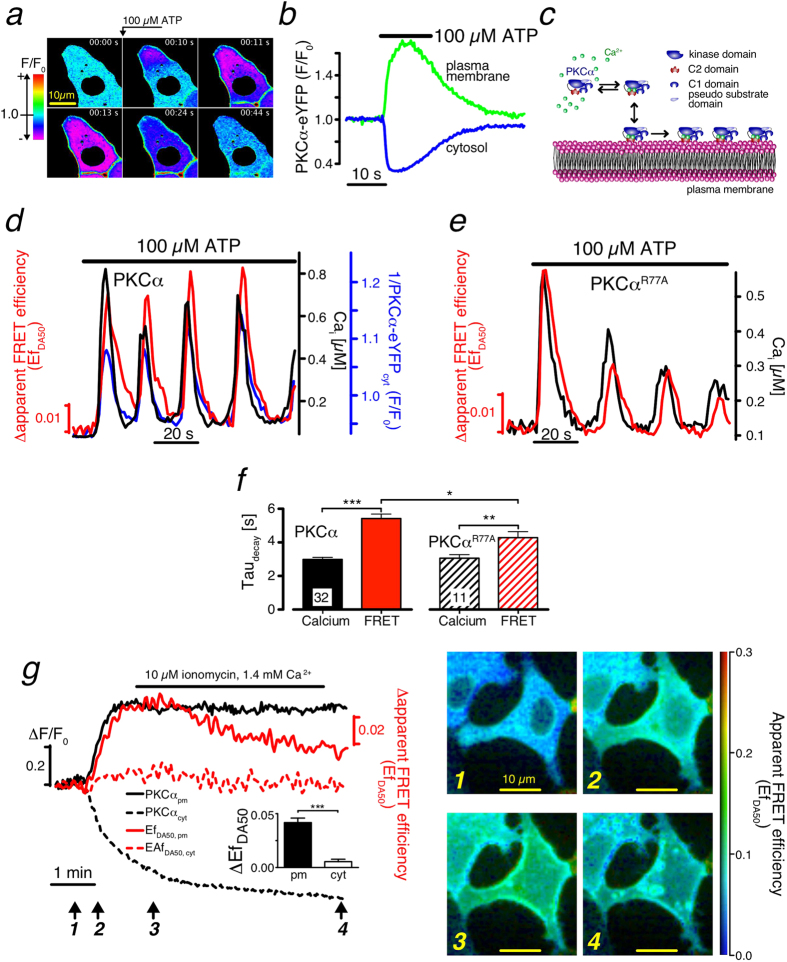
Experimental analysis of PKCα dynamics. (**a**) Time series of confocal images (F/F_0_) from a PKCα-eYFP expressing HEK cell stimulated with ATP (arrow) depicting plasma membrane accumulation. (**b**) Time course of the plasma membrane (green) and cytosolic (blue) PKCα-eYFP fluorescence during ATP stimulation. (**c**) Current molecular view of Ca^2+^ dependent PKCα translocation with independent PKCα molecules on the membrane leaflet. (**d**) ATP-induced Ca^2+^ oscillations (black) evoke PKCα-eYFP translocations from the cytosol (blue) and FRET between PKCα-eYFP and -eCFP (red). For easier reading the reverse cytosolic fluorescence (1/PKCα-eYFP_cyt_) has been plotted. (**e**) Ca^2+^ and FRET oscillations in a HEK cell expressing the mutant PKCα^R77A^ characterized by a substantially decreased DAG affinity. (**f**) Decay time constant of the resulting Ca^2+^ (black) and FRET transients (red) for HEK cells expressing PKCα (filled bar) and or the PKCα^R77A^ mutant (hashed bars). (**g**) Confocal recordings of PKCα translocation (black) and FRET changes (red) for the cytosol (dashed lines) and the plasma membrane (solid lines). Confocal FRET images are illustrated for the time points marked with arrows.

We hypothesized that dynamic, transient homo-protein intermolecular interactions of membrane-bound cPKC ensure increased signaling efficiency. To test this hypothesis, we studied initial steps of cPKC activation in living cells. To this end, we stimulated HEK cells expressing PKCα-eYFP with Adenosine-Triphosphate (ATP) that resulted in accumulation of the fusion-protein on the plasma membrane within a few seconds (Fig. 1a,b). The underlying mechanism is established and involves P2Y-receptor activating Gq-proteins and subsequent release of Ca^2+^ from internal stores^15^. Binding of Ca^2+^ to the C2-domain of PKCα increases its affinity for the inner leaflet of the plasma membrane, notably to negatively charged phospholipids such as phosphatidylserine (PS) (Fig. 1c)^16^,^17^. We used Förster resonance energy transfer (FRET) to probe the existence of intimate PKCα-PKCα interactions that could potentially increase lifetimes of membrane-bound PKCα molecules. To this end, we evoked Ca^2+^ oscillations in HEK cells co-expressing PKCα-eCFP and PKCα-eYFP. In case an excited eCFP finds itself in close proximity to eYFP it can excite the latter, such that the intensity of yellow fluorescence increases at the expense of cyan fluorescence. We found that the Ca^2+^ were accompanied by oscillations in the apparent FRET efficiency Ef_DA50_ (Fig. 1d, Materials and Methods). Remarkably, these FRET transients showed a substantially prolonged decay compared to those of the underlying Ca^2+^ transients (Fig. 1d,f). Notably, the decay extension was 2s (Fig. 1f), which is significantly larger than could be expected from the lifetime of 150 ms measured *in vitro* for membrane-bound PKCα-Ca^2+^ complexes^7^.

To investigate whether this prolongation was due to DAG binding we employed a PKCα mutant (PKCα^R77A^) with substantially reduced DAG binding^18^,^19^ (Fig. 1e). A quantitative analysis showed a faster FRET decay for the mutant PKCα^R77A^ compared to wt, but it remained significantly slower than the underlying Ca^2+^ decay (Fig. 1f). Next we asked whether FRET changes could be observed when only cytosolic Ca^2+^ was increased. For this, we utilized ionomycin to modulate the intracellular Ca^2+^ concentration [Ca^2+^]_i_ through changes of the extracellular Ca^2+^ concentration [Ca^2+^]_o_. We found that FRET changes occurred following an increase of [Ca^2+^]_i_. These changes were only observed on the plasma membrane, but were absent in the cytosol (Fig. 1g). Consequently, Ca^2+^ increases alone are sufficient for inducing intermolecular interactions resulting in prolonged membrane residence times.

Remarkably, extended exposure of cells to increased [Ca^2+^]_o_, resulted in an overshoot of the FRET efficiency, which eventually decayed to its steady state value (Fig. 1g, S1). It is important to note, that this decay occurred in the absence of changes of both, plasma-membrane bound PKCα levels and [Ca^2+^]_i_ (Fig. 1g, S1). The spontaneous FRET decay by 60% (Fig. S1) indicated that the main contribution to FRET resulted from specific intermolecular interactions between PKCα molecules (Fig. S2). We will address a possible contribution of molecular crowding below.

### A computational analysis of PKCα explains spontaneous FRET decay

**Figure 2:**
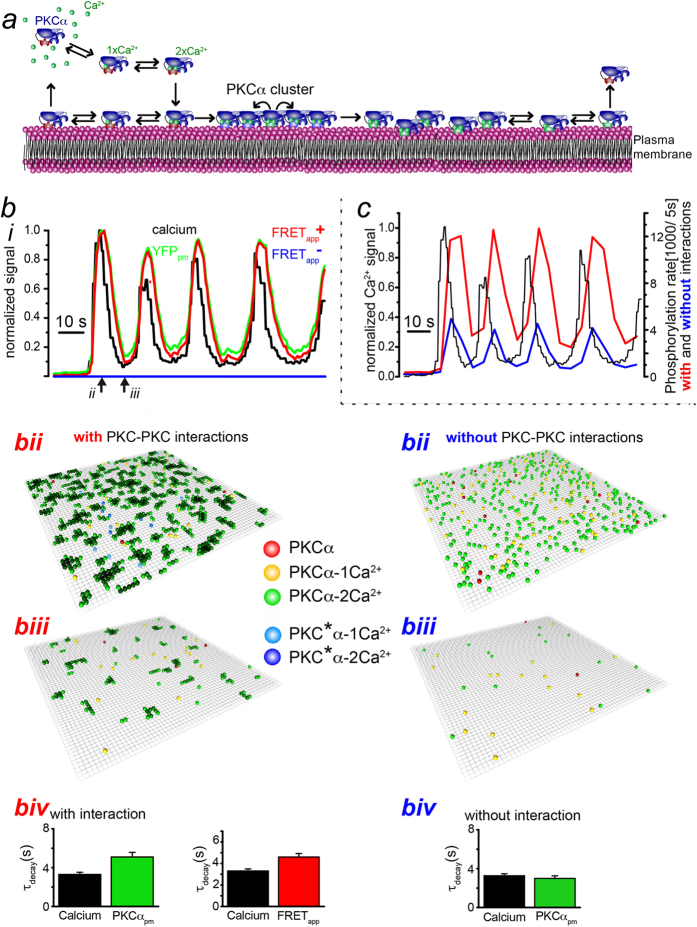
Theoretical analysis of PKCα dynamics. (**a**) Illustration of the molecular processes incorporated into our model. (**b**) Simulation results of PKCα behavior and FRET_app_ for the experimental Ca^2+^ signals of Fig. 1d with (red) and without (blue) intermolecular interactions. (ii, iii) Simulation snapshots for the time points indicated in (i). Color code for the snapshots in (ii) and (iii): red, yellow, and green represent PKCα in state 1 with 0, 1 and 2 bound Ca^2+^, respectively. Darker green indicates smaller Ca detachment rates. The green color of membrane-bound particles gets darker with the number of bound neighbors. Light and dark blue represent PKCα in state 2 with 1 or 2 bound Ca^2+^, respectively. (iv) Characteristic decay times of PKCα at the membrane (green) and corresponding FRET_app_ (red) averaged over N = 18 simulation runs. Simulation parameters as given in Table 1. (**c**) Simulation of PKCα-dependent phosphorylation for the experimental Ca^2+^ signals of Fig. 1d with (red) and without (blue) assuming intermolecular interactions.

In order to further explore the mechanistic consequences of intimate PKCα interactions, we performed a computational analysis of PKCα dynamics accounting for the process of PKCα aggregation (Fig. 2s, Materials and Methods). Our model is based on the key assumption of adjacent PKCα molecules to constitute intimate links resulting in the formation of stable clusters on the plasma membrane (Fig. 2s). In the model, we consider that each PKCα-molecule has two Ca^2+^-binding sites (a single C2 domain is known to coordinate 2 Ca^2+^ ions). In the cytosol, they are occupied independently at a rate 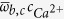, where 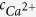 is the concentration of Ca^2+^ that we take to be spatially homogenous. Unbinding of Ca^2+^ from the two sites occurs independently at rate 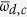. Only when both binding sites are occupied, PKCα binds to the membrane at rate *ω*_*a*_.

From the observation that the FRET signal decayed in presence of a constant fluorescence signal, we inferred that membrane-bound PKCα can be in two states: in state 1 it can form a cluster, such that it is tightly bound to the membrane and neighboring molecules. In state 2, it cannot form a cluster. In our model, transitions from state 1 to state 2 take place within the cluster at rate *ω*_*t*_ and are irreversible. The unbinding rate of Ca^2+^ depends on the state of PKCα: In state 1, the rate depends on the number *n* of neighboring molecules 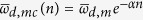, whereas in state 2 it is 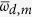. Membrane-bound PKCα can bind Ca^2+^ at a free Ca^2+^-binding site at rate 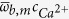. Once a PKCα-molecule has lost both Ca^2+^ it detaches from the membrane at rate *ω*_*d*_.

Motivated by the rapid dissociation of PKCα clusters observed for local translocation events^19^, we assume furthermore that once an individual molecule has switched to the second, less stable state it induces such transitions in neighboring molecules at rate *ω*_*it*_. These transitions spread rapidly through the clusters and destabilize them. The lifetime of a PKCα cluster is thus determined by the rate of transitions from state 1 to state 2 and by the total number of molecules in the cluster.

We employed particle-based stochastic simulations to explore the dynamic behavior of this system. The parameter values used in our simulations were taken from the literature whenever they existed, whereas the remaining values were chosen such that our simulation results matched those from our experiments (Table 1). First, we used the recorded Ca^2+^ oscillations depicted in Fig. 1d to compute the expected apparent FRET efficiency by counting the number of neighboring PKCα on the membrane (FRET_app_, Fig. 2b). The computed FRET_app_ transients showed the same prolonged decay as the measured FRET signal, when compared to the Ca^2+^ transient (Fig. 2b,f, Movie 1). Secondly, we computed Ca^2+^ dependent phosphorylation in the presence of PKCα-PKCα interaction (red traces in Fig. 2c) or in its absence (blue trace in Fig. 2c). To this end, we assumed that all membrane-bound PKCα-molecules were active and phosphorylated at a constant rate *ω*_*p*_. To obtain a total phosphorylation rate, we counted all phosphorylation events in subsequent time windows of 5 s length. The corresponding data showed that intermolecular interactions increased the total phosphorylation rate by a factor of almost 3 (Fig. 2c, Figure S3).

### Synchronization of membrane-bound PKCα

**Figure 3:**
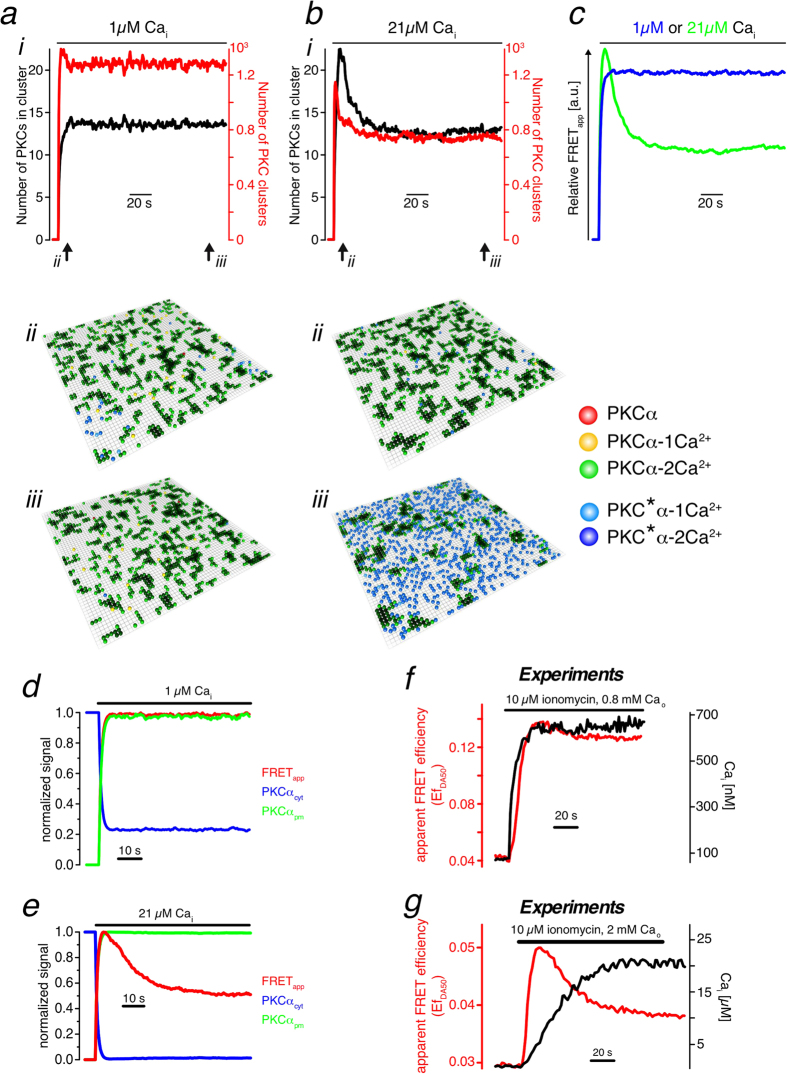
Molecular mechanism of spontaneous FRET decay. (**a,b**) (i) Mean cluster size (black) and number of clusters (red) during prolonged [Ca^2+^]_i_ increases for [Ca^2+^]_i _= 1 μM and [Ca^2+^]_i_ = 21 μM. (ii, iii) Simulation snapshots for the time points indicated in (i). (**c**) FRET_app_ efficiency from (**a**) and (**b**) for high and low [Ca^2+^]_i_. (**d**–**g**) Spontaneous FRET decay during prolonged [Ca^2+^]_i_ increases for [Ca^2+^]_i_ ≈ 1 μM (**d,f**) and [Ca^2+^]_i_ ≈21 μM (**e**,**g**). Traces in (**d,e**) result from simulations, in (**f,g**) from experiments.

We used our model to analyze the dynamics of PKCα clusters during prolonged increases of [Ca^2+^]_i_ (Fig. 3). For [Ca^2+^]_i_ ≈ 1 μM the mean cluster size and the number of clusters increased monotonically with time (Fig. 3a, Movie 2). On average clusters consisted of around 13 molecules. Incidentally, this cluster size is very similar to the size of clusters providing optimal fidelity in digital signalling as reported in a theoretical study^31^. For higher Ca^2+^ concentrations, [Ca^2+^]_i_ ≈ 20 μM, and after long exposure to an increased Ca^2+^ concentration, the mean number of molecules in a cluster was essentially the same as for the lower Ca^2+^ concentration (Fig. 3b, Movie 3). The corresponding number of clusters was lower. A higher Ca^2+^ concentration leads to a higher net rate of PKCα binding to the membrane and thus to larger clusters. Their lifetime is shorter than that of smaller clusters. Consequently, the average number of clusters on the membrane is reduced.

Much more strikingly than the change in the number of clusters in steady state was, however, that the mean cluster size and the total number of clusters now displayed a pronounced peak before decaying to their steady state values. These data suggest that the global spontaneous FRET decay observed in living cells (Fig. 1g, S1) was a result of localized cluster formation and dissociation dynamics.

Corresponding calculations of the system’s total FRET_app_ efficiency confirmed a spontaneous decay for the higher [Ca^2+^]_i_ (Fig. 3c–e). Strikingly, data from living cells matched this prediction (Fig. 3f,g). Moreover, there is a strong positive correlation between the spontaneous FRET decay and [Ca^2+^]_i_ for a wide range of concentrations (Fig. S4a) whose correlation level was substantially above the coincidence level (Fig. S4b). From these data we conclude that upon [Ca^2+^]_i_ increases larger than about 5 μM, PKCα molecules bind essentially simultaneously to the plasma membrane and form nano-clusters in a synchronized manner. Subsequent cluster dissociation and formation is increasingly desynchronized such that the FRET efficiency gradually decays, eventually reaching a steady state.

### C2 domain is responsible for nano-cluster formation

**Figure 4:**
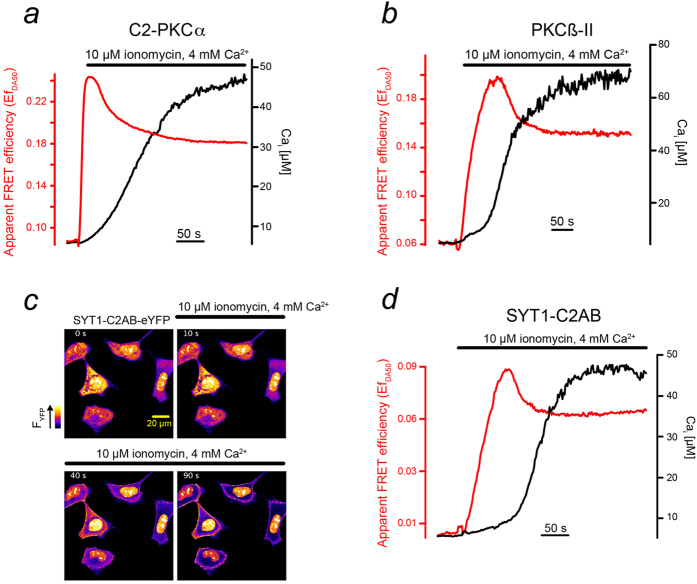
Ca^2+^ induced clustering of C2-domain containing proteins. (**a,b,d**) Prolonged [Ca^2+^]_i_ (black) induced increased FRET signals (red) with a peak and subsequent decay for the isolated C2-domain of PKCα (**a**), for PKCβ-II (**b**) and a truncated version of synaptotagmin (SYT1-C2AB) (**d**). (**c**) In response to a [Ca^2+^]_i_ increase SYT1-C2AB-eYFP accumulates at the plasma membrane. Series of confocal images at the time points given.

We wondered further, which domain of the PKCα molecule was essential for mediating intermolecular FRET. Because PKCα did essentially not aggregate in the cytosol (Fig. 1g), we speculated that the domain responsible for aggregation is the Ca^2+^ binding C2-domain itself. We thus expressed C2-domains of PKCα fused to eCFP ((PKCα) C2-eCFP) and eYFP and investigated possible FRET changes upon a Ca^2+^ increase (Fig. 4a). Following the rise in Ca^2+^, C2-domains rapidly translocated to the plasma membrane (Figure S5a). Extended elevated [Ca^2+^]_i_ resulted in a rapid increase in FRET that peaked and eventually leveled out to a new plateau value (Fig. 4a), a behavior that resembled that of the full length PKCα molecule (see Figs 1.g and 3.g).

To further substantiate our notion, we used another important member of the cPKC subfamily, PKCβ-II, involved, for example, in insulin secretion and immune responses^3^,^20^. In contrast to the C2-domain of PKCα with two Ca^2+^ binding sites the C2-domain of PKCβ-II contains three Ca^2+^ binding sites^5^,^7^,^21^. In cells co-expressing PKCβ-II-eCFP and PKCβ-II-eYFP, we observed fast translocation to the plasma membrane (Fig. S5b), the characteristic peak, and the subsequent decay to a plateau value of the FRET-efficiency (Fig. 4b). We concluded that the formation of nano-clusters might be a universal feature of Ca^2+^ sensing proteins using C2-domains.

To test this hypothesis, we employed SNARE proteins constituting a family of Ca^2+^ sensing proteins that is structurally and functionally unrelated to PKCs. They play an essential role in vesicle fusion by mediating an important Ca^2+^ dependent step in the exocytotic mechanism^22^. We focused on Synaptotagmin-1 (SYT1), which contains a tandem C2-domain^23^,^24^. *In vitro*, the second C2 domain of synaptotagmin was found to induce synaptotagmin oligomerization^25^. Since SYT1 comprises a Ca^2+^-independent lipid-anchoring domain, SYT1 is localized to the plasma membrane already under resting conditions. We thus constructed a novel protein, SYT1-C2AB that lacked lipid anchoring and thus resides in the cytosol under resting conditions (Fig. 4c). Increases in [Ca^2+^]_i_ resulted in a rapid and substantial translocation of SYT1-C2AB from the cytosol to the plasma membrane (Fig. 4c). Concomitant with the Ca^2+^ increase, the apparent FRET efficiency peaked and subsequently decayed towards its steady-state value (Fig. 4d). These data further support our conclusion that the formation of nano-clusters is a universal feature of C2-domain containing proteins upon Ca^2+^-dependent membrane binding.

### Molecular crowding does not contribute to FRET increases

After translocation to the plasma membrane, two different mechanisms can lead to increases in FRET: either specific intramolecular interactions leading to the formation of nano-clusters or molecular crowding, that is, a high membrane density of the proteins resulting in unspecific interactions. To estimate the possible contribution of molecular crowding to the FRET changes observed in this study we determined the correlation between the FRET change and the total cellular fluorescence intensity for each image pixel during the entire experiment (Fig. S2). A positive correlation between these two parameters could not be detected. If crowding were a main contributor to the observed FRET changes, the positive correlation should even increase during accumulation of PKCα at the plasma membrane. Such an increase was not present in our data (Fig. S2).

**Figure 5:**
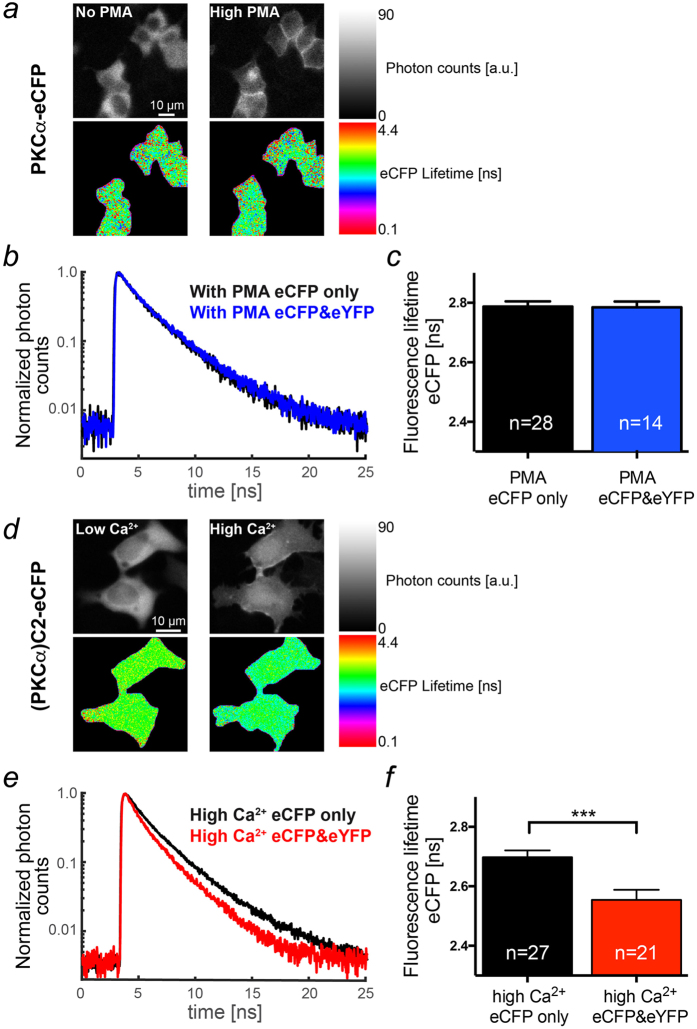
Ca^2+^ but not PMA mediated membrane accumulation induces nano-cluster formation. (**a**) Distribution of fluorescence intensity (upper row) and lifetime (lower row) for PKCα-eCFP before (left column) and following PMA treatment (right column, 1 μM, 20 minutes) acquired by time-correlated single photon counting (TCSPC). (**b**) Exemplified typical normalized fluorescence decay histograms of PKCα-eCFP acquired from HEK cells expressing either PKCα-eCFP alone (black) or PKCα-eCFP and -eYFP (blue). (**c**) Statistical analysis of the eCFP lifetime in populations of HEK cells treated as described for (**a**). (**d**) Distribution of fluorescence intensity (upper row) and lifetime (lower row) for the C2-domain of PKCα ((PKCα)C2-eCFP) before (left column) and following permeabilization with ionomycin (10 μM) (right column, Ca_o_ = 1.8 mM, 5 minutes) acquired by TCSPC. (**d**) Exemplified typical normalized fluorescence decay histograms of (PKCα)C2-eCFP acquired from HEK cells expressing either (PKCα)C2-eCFP alone (black) or (PKCα)C2-eCFP and -eYFP (red). (**e**) Statistical analysis of the CFP lifetime in populations of HEK cells treated as described for (**d**).

To scrutinize our findings, we studied the fluorescence lifetime of eCFP fusion proteins at the plasma membrane in the absence and presence of appropriate FRET partners, i.e. eYFP fusion proteins. In order to maximize accumulation of PKCα molecules on the plasma membrane in the absence of Ca^2+^ increases, we employed the phorbol ester phorbol 12-myristate 13-acetate (PMA) well-known to cause maximal PKCα translocation^26^. The PMA treatment resulted in substantial plasma membrane accumulation of PKCα (Fig. 5a, upper row of images) and could thus provoke molecular crowding and/or nano-cluster formation. The fluorescence lifetime of eCFP did not change in the presence of its principle FRET partner eYFP (Fig. 5b,c) indicating that despite a high plasma membrane prevalence of PKCα intermolecular interactions were not specific enough to cause increases in FRET.

In contrast, following Ca^2+^ induced translocation to the plasma membrane (Fig. 5d, upper row of images) the fluorescence lifetime of (PKCα)C2-eCFP was significantly reduced from 2.70 ± 0.02 ns in the absence of (PKCα)C2-eYFP, i.e. no FRET, to 2.54 ± 0.02 ns in its presence, i.e. with FRET (Fig. 5d,e). These data supported the results of our lux-FRET measurements, demonstrating specific intermolecular interactions and further demonstrated that nano-cluster formation of membrane-bound PKCα relies on its C2 domain.

While that membrane translocation is a prerequisite for nano-clustering, the molecular origin of C2-domain mediated interactions remains to be discovered. Only then one may be able to construct C2-domains that attach to the membrane in response to a Ca^2+^ increase and study the effect of other domains on clustering.

## Conclusion

We employed intermolecular FRET in living cells combined with computational analysis to study the spatial organization of cPKCs binding to the plasma membrane, a prerequisite for cPKC activation. We discovered transient Ca^2+^-dependent cPKC nano-clusters that significantly extend the plasma-membrane residence time of cPKC molecules. Such increases in the membrane residence time overcome the inherently slow phosphorylation rates of the PKCs´ kinase domain and result in more efficient downstream signaling. Stochastic simulations pointed to a 3-fold increased signaling efficiency of cPKCs in nano-clusters. These protein arrays resulted from cPKC self-assembly through their Ca^2+^-binding C2-domain, a molecular motif widely used for membrane targeting of Ca^2+^-sensing proteins^27^. We also established clustering of other unrelated C2-domain containing proteins and even of isolated C2 domains, suggesting that in living cells nano cluster formation is a general feature of Ca^2+^-dependent membrane-binding proteins utilizing C2-domains.

Our findings strongly indicate that nano-cluster formation of C2-domain containing proteins constitutes an essential step in Ca^2+^ readout during cellular signaling and emphasize the importance and versatility of such cooperative effects for the cellular signaling toolkit.

## Materials and Methods

### Cell Culture and Transfection

HEK293 cells were cultured as described previously^26^. 24 hours before transfection HEK293 cells were transferred into 20 mm glass coverslips. For transfection with the plasmids we used NanoJuice^®^ (Novagen, USA) according to the vendor´s recommendations. Cells were investigated 48 hours after transfection.

### Solutions

All experiments were conducted at room temperature (20–22 °C) and used an extracellular solution (Tyrode) comprising: 135 mM NaCl, 5.4 mM KCl, 2 mM MgCl_2_, 1.8 mM CaCl_2_, 10 mM glucose, 10 mM HEPES adjusted to pH 7.35 with NaOH unless stated otherwise. All compounds used were of research grade. Changes in the extracellular solution were achieved by a gravity-driven custom-made local perfusion system or by manually exchanging the bath solution.

For long-term changes in the intracellular Ca^2+^ concentration, we made use of the Ca^2+^ -ionophore ionomycin (Sigma, Germany) and adapted the extracellular Ca^2+^ concentration as detailed in the figures. Extracellular Ca^2+^ concentrations were modulated simultaneously with the application of the ionophore. Please note that this experimental design could result in varying speeds of Ca^2+^ changes due to the varying efficiency, with which ionomycin incorporated into the plasma membrane. The resulting changes in the intracellular Ca^2+^ concentration were quantified after pre-loading the cells with Indo-1 or Mag-Indo-1 (see below).

### Plasmids (Fluorescence-labeled protein)

The wild-type human PKCα protein, C1 domain mutated (R77A) PKCα protein and full length of human PKCβII protein were fused with eYFP or eCFP at the C-terminus in the pCDNA3 plasmid as described previously^19^.

The C2 domain of PKCα was cloned from human PKCα by PCR with the following primers, 5′-AGAATTCATGGATCACACTGAGAAGAGG and 5′-ATCTCGAGCGGTCCGTGAGTTTCACTCG, and fused with eYFP or eCFP at the C-terminus respectively in the pCDNA3 plasmid.

The truncated form of synaptotagmin-1 (SYT1-C2AB) was cloned from the full length of synaptotagmin-1 (EMBL) with the following pair of primers, 5′-ATCTCGAGAGAAATGTTTGTTCAAAA and 5′- TGGATCCTACTTCTTGACGGCCAG, and fused with eYFP or eCFP respectively at the N-terminus in the pCDNA3 plasmid.

All plasmids were confirmed by sequencing.

### Video Imaging

For combined FRET and Ca^2+^-imaging employing Indo-1 or MagIndo-1 we utilized an automated inverted microscope (uiMic, TILL Photonics, Germany) and a monochromator (Polychrome V, TILL Photonics, Germany) for generating the required excitation light (Indo-1/MagIndo-1: 350/5 nm; eCFP: 430/5 nm and eYFP: 512/5 nm). The resulting pairs of images were projected on the two halves of a fast CCD camera (Retiga-2000R, Qimaging, Canada) after passing through appropriate dichroic mirrors (Indo-1/MagIndo-1: 465/15 nm; eCFP/YFP: 542/20 nm). Imaging was performed through a 20x multiimmersion lens (UPLSAPO oil NA 0.85, Olympus, Germany). The entire setup was controlled by LA software (version 2.2.0.12, TILL Photonics, Germany). Before the experiments with combined FRET and Ca^2+^ measurements were started the HEK293 cells on the coverslip were loaded with either Indo-1 AM (0.5 μM) or MagIndo-1 AM (0.5 μM) in Tyrode for 30 minutes. Thereafter the cells were bathed in normal Tyrode without the dye for de-esterification (20 minutes). To perform the calculation of the FRET efficiency and the Ca^2+^ concentration, we acquired three pairs of images; two image pairs CFP/YFP at the CFP excitation and YFP excitation wavelengths and one image pair at the Indo-1, MagIndo-1 excitation wavelength. Images acquired in a frequency of 0.5 fps with image size 200 × 300 pixels.

### Confocal Imaging

We performed confocal imaging on an inverted microscope (TE-2000E, Nikon, Germany) using an oil immersion objective (40x, NA 1.3 S-Fluor, Nikon, Germany). The microscope was attached to a fast 2D-kilobeam array scanner (Infinity-3; VisiTech Int., UK) that simultaneously scans 2500 parallel laser beams across the specimen and projects the resulting fluorescence images on two spectrally separated EMCCD-cameras (iXon 887, Andor Technology, UK). For alternating excitation of eCFP and eYFP (see below) we employed two solid-state lasers; eCFP excitation with a 445 nm laser (Toptica, Germany), eYFP excitation with a 514 nm laser (Cobolt, Sweden). The two emission channels were separated through a dichroic mirror (491 nm). The entire setup was integrated and controlled through VoxCellScan software (VisiTech Int., UK). To perform the calculation of the FRET efficiency, we acquired two pairs of images CFP/YFP at the CFP excitation and YFP excitation wavelengths. Images (256 × 256 pixels) were recorded at 0.5 fps.

### Data handling

After the experiments, the resulting image series were transferred into a large-scale image database system running OMERO 5.02 (Open microscopy environment, University of Dundee, UK) for long-term storage. Images were processed either in MatLab (see below) or in ImageJ. To obtain fluorescence over time plots, the fluorescence information from regions-of-interests were averaged, saved and imported into Igor software (Wavemetrics, USA).

Where appropriate, we calculated so-called self-ratio traces or images (F/F_0_), for which the fluorescence at a given time point (F) was divided by the resting fluorescence (F_0_) to account for different dye loading and/or expression of the fluorescent proteins and their distribution in subcellular compartments (see also ref. 19).

Final figure design was performed with Adobe Illustrator CS6 (Adobe, USA).

### Spectral lux-FRET analysis

To investigate cPKC nano-clusters we performed quantitative lux-FRET experiments according to Wlodarczyk *et al*.^28^. Apparent FRET efficiencies *Ef*_*D*_ and *Ef*_*A*_, which are the absolute FRET efficiencies scaled by the respective fractions of donors and acceptors in the FRET state and the donor mole fraction (*x*_*D*_ = [*D*^*t*^]/([*D*^*t*^] + [*A*^*t*^])), were determined for each pixel for each FRET measurement. Here [*D*^*t*^] and [*A*^*t*^] denote total donor and acceptor concentrations, respectively. Data stacks acquired at alternating excitation wavelengths (445 nm and 514 nm) were obtained with two emission images each (see confocal imaging setup), corrected for background, inhomogeneous illumination, and further processed according to Prasad *et al*.^29^. To compare the apparent FRET efficiencies at varying *x*_*D*_ values, we calculated the predicted apparent FRET efficiency 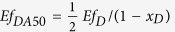
at equal donor and acceptor concentrations (*x*_*D*_ = 0.5), assuming a standard dimerization model^30^. All processing was performed with custom made Matlab scripts.

### Fluorescence lifetime imaging

Fluorescence lifetime microscopy (FLIM) images were acquired with a custom made time-correlated single photon counting (TCSPC) system comprising an inverted microscope (Nikon TE-2000-U), a super-continuum laser (SC430-4, Fianium Ltd., Southampton, UK) with a repetition rate of 40 MHz coupled into a laser scanning unit (Yanus IV, FEI Munich GmbH, Germany). The setup was controlled by image acquisition software (LA, Version 2.5, FEI Munich GmbH). The sample was scanned using a 40x, 1.4 NA Plan-Fluor objective, creating images of the size 384 × 384 pixel. The start and stop signals for each line of the image were transferred from the scanning unit into the TCSPC system (HydraHarp, Picoquant GmbH, Berlin, Germany) for constructing the resulting image. Emitted photons were detected by an avalanche photodiode (APD), acquired, time-stamped and analyzed by the TCSPC Software (Symphotime64, Picoquant GmbH, Berlin, Germany). Excitation of eCFP was carried out through a bandpass filter (434/17 nm) and the emission was detected through a bandpass filter (475/42 nm, AHF Analysentechnik AG, Tübingen, Germany).

### Computational model of PKCα-dynamics

In our computational model, PKCα-molecules are represented by particles with a diameter of 30 nm moving on a cubic lattice with an edge of length 7.2 μm × 7.2 μm × 1.8 μm representing the cytosol. Sites have a lateral extension equaling a particle diameter. The membrane is represented by a square lattice that coincides with one of the faces of the cubic lattice introduced before. We apply periodic boundary conditions in the directions lateral to the membrane.

We consider three states of PKCα: Ca^2+^-free PKCα molecules, PKCα bound to one and to two Ca^2+^ ions. Unbound Ca^2+^ ions are assumed to form a reservoir. Binding of a Ca^2+^ ion to PKCα occurs at rate ω_b,c_[Ca^2+^]_i_, where [Ca^2+^]_i_ is the (intracellular) Ca^2+^ concentration. PKCα release Ca^2+^ ions at rate ω_d,c_. Particles can hop to empty neighboring sites at constant rate corresponding to a diffusion constant D_c_.

The membrane is described by a 7.2 μm × 7.2 μm square lattice that coincides with one face of the cube. PKCα bound to two Ca^2+^ ions can attach to the membrane at rate ω_a_ (technically they switch from a site of the cubic lattice representing the cytosol to the co-localized site on the square lattice representing the membrane). Particles on the membrane exist in one of three states corresponding to PKCα that is not part of a cluster and to two conformations of PKCα when it is part of a cluster. The detachment of Ca^2+^ from an isolated PKCα occurs at rate ω_d,m_. Ca^2+^ binds to membrane-bound PKCα at rate ω_b,m_[Ca^2+^]_i_. PKCα free of Ca^2+^ detaches from the membrane at rate ω_d_.

Two adjacent PKCα molecules on the lattice are assumed to strongly interact and to immediately form a cluster. A particle that has not been part of a cluster before changes its state to the stable cluster state. For PKCα in this state, the interactions between PKCα and Ca^2+^ are stabilized. The rate of Ca^2+^ release then depends on the number n of adjacent PKCα in the cluster and equals ω_d,mc_(n) = ω_d,m_ exp{−αn}.

Ca^2+^-ions bind to membrane-bound 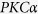 with the rate ω_b,m_ [Ca^2+^]_i_. 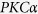 molecules in a cluster undergo spontaneous conformation switches with the rate ω_t_. Proteins in the new conformation are denoted as 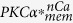. Molecules in the new conformation change the transition rate of their neighbouring molecules from ω_t_ to ω_it_. 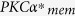 molecules cannot self-assemble into clusters any longer and they release Ca with the rate ω_d,m_.

The particles obey the following reactions occurring stochastically. The actual values for the rates of the reactions are given in Table 1.


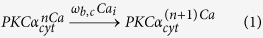



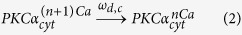



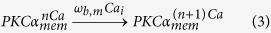



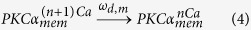



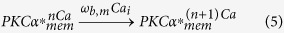



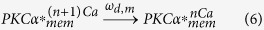



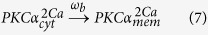



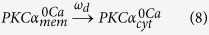



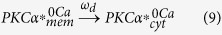



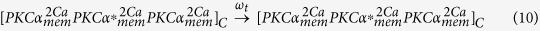



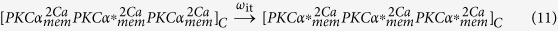


We analyze the dynamic behavior of 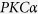 molecules taking into account all of the considered reactions by using a particle-based Monte Carlo simulation based on the Gillespie algorithm. 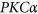 molecules are simplified by spherical particles with a diameter of 0.03 μm. They are in a cuboid-shaped domain of size 7.2 μm × 7.2 μm × 1.8 μm, consisting of a three dimensional bulk and a two dimensional membrane. The bulk and the membrane are represented by a 3d cubic lattice and a 2d square lattice, respectively. Particles can only move from one lattice point to an adjacent lattice point. The distance between two lattice points is 0.03 μm. The diffusion constant of particles in the bulk D_c_ and the diffusion constant of membrane-bound particles D_m_ differ by a factor of 10. At the system’s boundaries we apply periodic boundary conditions. Only particles which diffuse in the bottom layer of the bulk can bind to membrane. At the top of the geometry, we apply no flux boundary conditions. The clusters are formed when membrane-bound 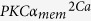 molecules come in contact. In clusters, the particles are fixed and they cannot diffuse any longer.

In the simulations we do not model the fluorescence explicitly. Fifty percent of the particles are marked with the label ‘YFP’, all the rest is marked with the label ‘CFP’. ‘CFP’- and ‘YFP’-labelled particles have exactly the same molecular properties. The total FRET signal is defined as the number of ‘CFP’-labelled particles in a complex with at least one adjacent ‘YFP’-labelled particle.

### Statistics

Statistical analysis of the data was performed in Prism 6 software (GraphPad, USA). After testing for Gaussian distribution (D’Agostino-Pearson omnibus normality test) data were analyzed with an unpaired t-test. Bar graphs depict mean ± SEM. Statistical significance was defined as follows; *p < 0.05, **p < 0.01, ***p < 0.001. N numbers give the number of cells analyzed, whereas the number of experiments depict independent experiments (different passage or cover slip).

## Additional Information

**How to cite this article**: Mike, B. *et al*. C2-domain mediated nano-cluster formation increases calcium signaling efficiency. *Sci. Rep.* **6**, 36028; doi: 10.1038/srep36028 (2016).

**Publisher’s note**: Springer Nature remains neutral with regard to jurisdictional claims in published maps and institutional affiliations.

## Supplementary Material

Supplementary Movie 1

Supplementary Movie 2

Supplementary Movie 3

Supplementary Information

## Figures and Tables

**Table 1:** Parameter and their values for the stochastic simulation.

*D*_*c*_	*D*_*m*_	*ω*_*b,c*_	*ω*_*b,m*_	*ω*_*d,c*_	*ω*_*d,m*_	*ω*_*it*_	*ω*_*a*_	*ω*_*d*_	*ω*_*t*_	*α*
10 *μm*^*2*^*/s*	1 *μm*^*2*^*/s*	25 · 10^7^ 1/*M*_*S*_	5 · 10^7^ 1/*Ms*	65 1/*s*	16 1/*s*	200 1/*s*	100 1/*s*	100 1/*s*	0.003 1/*s*	1.4
Schaefer *et al*. (2001)	Lippincott-Schwarz *et al*. (2001)	Kohout (2002), Reither *et al*. (2006), Nalefski (2001), Dekker, Protein Kinase C, Kluwer publishing				estimated				

Protein density was 250 μm^−3^.
