# Supplementary material for: C2-domain mediated nano-cluster formation increases calcium signaling efficiency
Source: Sci Rep. 2016 Nov 3;6:36028. doi: 10.1038/srep36028 (PMC5093555; doi:10.1038/srep36028)
Supplement: Supplementary Information [file srep36028-s4.pdf]

Title: C2-domain mediated nano-cluster formation increases calcium signaling efficiency

Authors: Mike Bonny[1§], Xin Hui[2§], Julia Schweizer[2], Lars Kaestner[2], André Zeug[3], Karsten Kruse[1*], Peter Lipp[2*]

Affiliations:

[1]Theoretical Physics, Saarland University, Saarbrücken, Germany.

[2]Institute for Molecular Cell Biology, Medical Faculty, Saarland University, Homburg/Saar, Germany.

[3]Cellular Neurophysiology, Center of Physiology, Hannover Medical School, Hannover, Germany.

[§]These authors contributed equally to this work.

[*]Shared senior authorship.

Correspondence to: peter.lipp@uks.eu or k.kruse@physik.uni-saarland.de

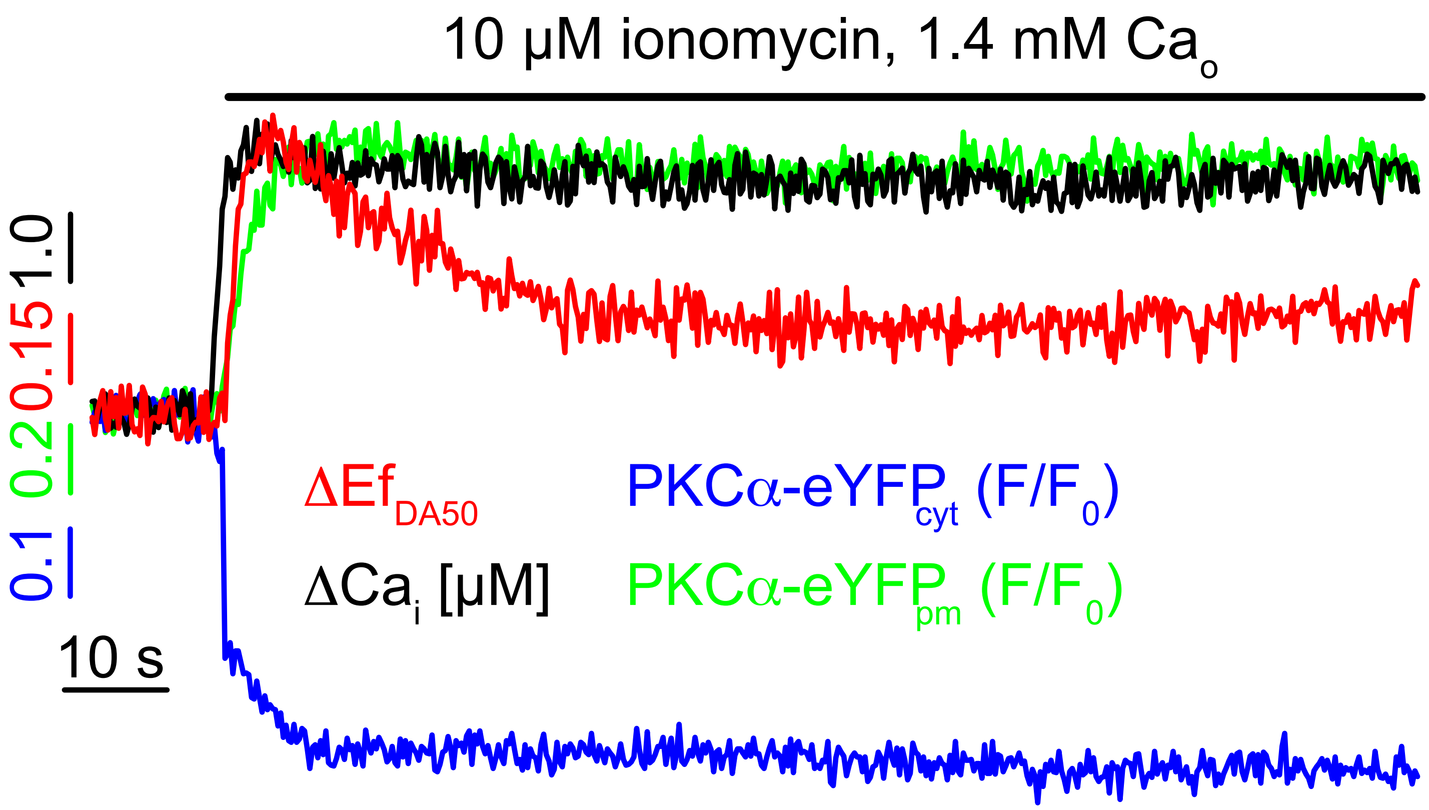

Spontaneous FRET decay in the presence of prolonged increases in [Ca^{2+}]$_i$. After an initial peak in FRET (red) spontaneous FRET decrease occurs eventually resulting in a new steady state value. Note, that this FRET decay occurs in the presence of a constant intracellular Ca concentration (black trace) and maintained translocation of PKCα to the plasma membrane (green).

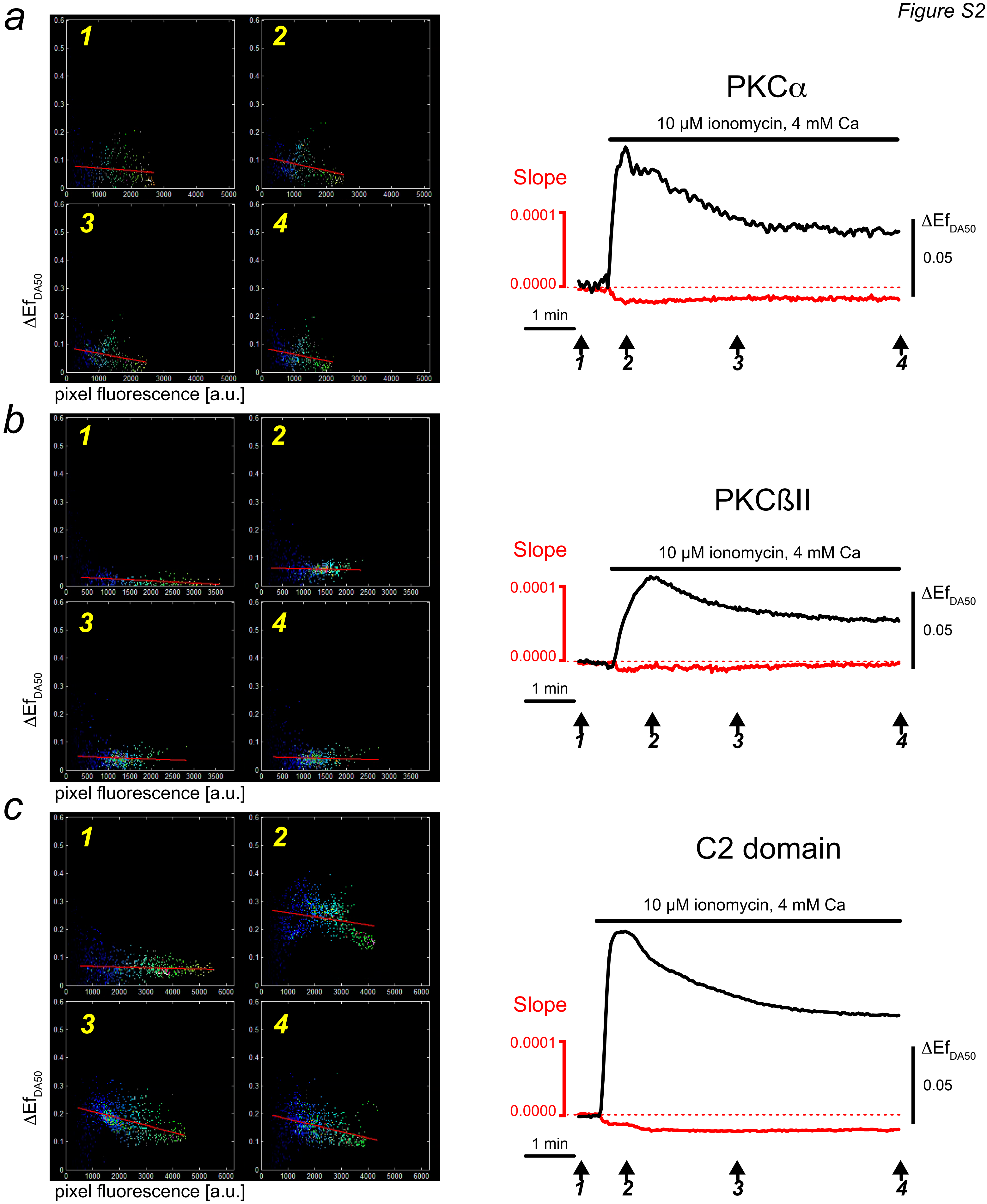

Molecular crowding is not at the origin of the increased FRET efficiency after Ca^{2+}-induced membrane translocation. Left column presents the FRET efficiency Ef_{DA50} as a function of the pixel fluorescence intensity for HEK cells expressing the CFP and YFP fusion proteins of PKCα(a), PKCßii (b), and C2-domain of PKCα (c). The pixel fluorescence intensity is given as the geometric mean of the donor and the acceptor emission intensities. In each case, distributions acquired at four different times for representative cells are shown. The color of the dots indicates the frequency at which they occurred with warmer color corresponding to higher frequencies. The data were acquired for all pixels in the respective cell regions. Red lines indicate the results of a linear fit. Molecular crowding as the principal reason for the increased FRET efficiency would lead to a positive correlation. Right column: FRET efficiency Ef_{DA50} as a function of time for the cells shown on the left (black curves) and slopes of the linear fits to the data points (red). Black arrows and numbers indicate the selected time points shown on the left. In all cases, the FRET efficiency spontaneously decays and presents a negative correlation with the pixel intensity. Together these results strongly indicate that the increase of the FRET efficiency upon stimulation of membrane translocation is not primarily due to molecular crowding

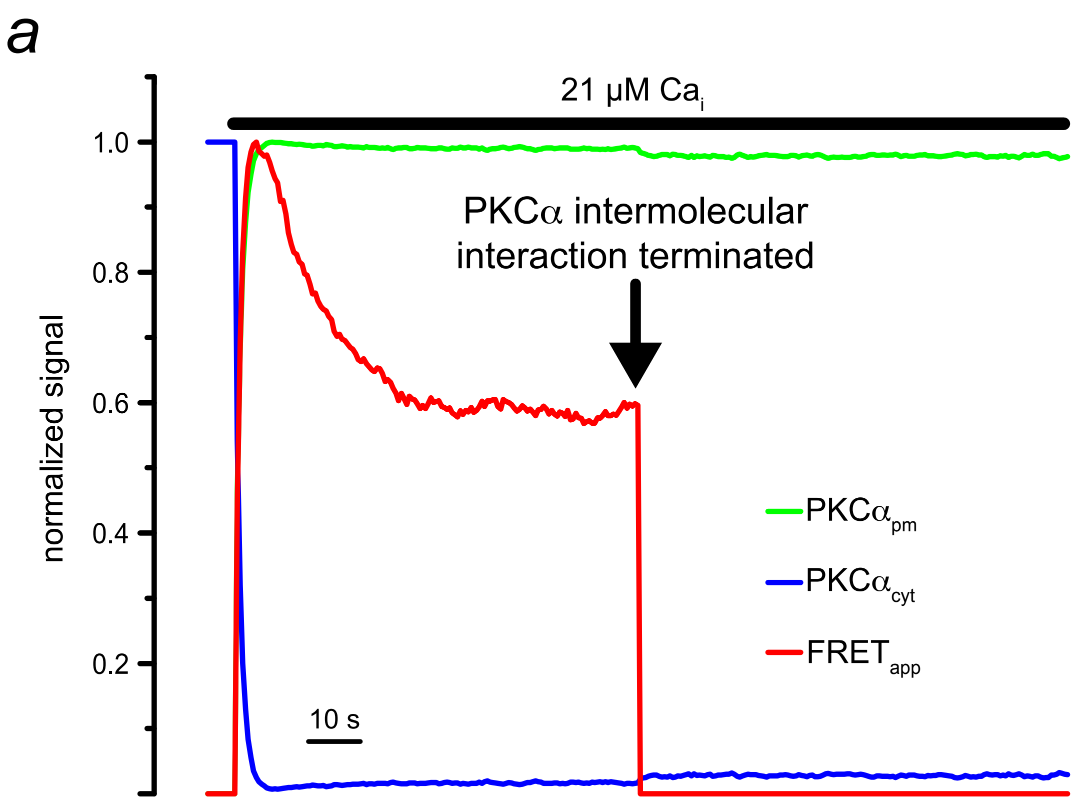

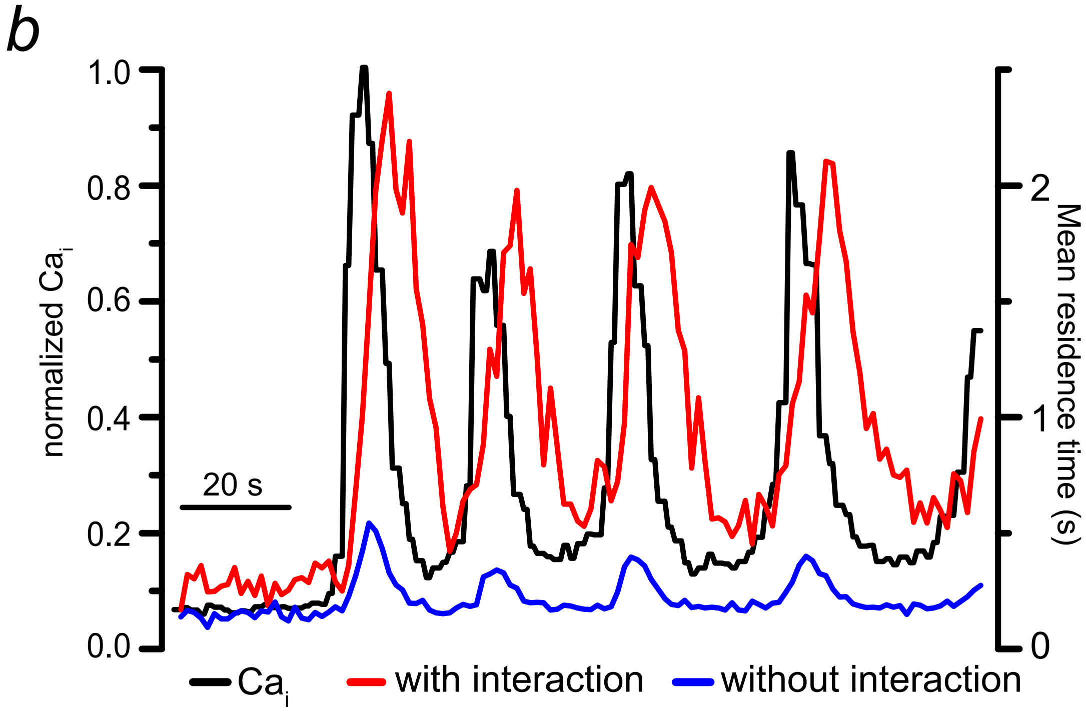

Comparison of simulation results with and without interparticle interactions. (a) Concentration of membrane-bound PKCα (green curve), cytosolic PKCα (blue), and the total $FRET_{app}$ signal (red) for a simulation of an extended elevated Ca_i concentration of 21µM. After 1min, interparticle interactions were switched off. (b) Mean residence time of membrane-bound PKCα with (red curve) and without (blue) interparticle interactions from simulations for the measured oscillatory Ca_i signal (black) from Fig. 1d (main text). Parameters are as in Table 1.

a

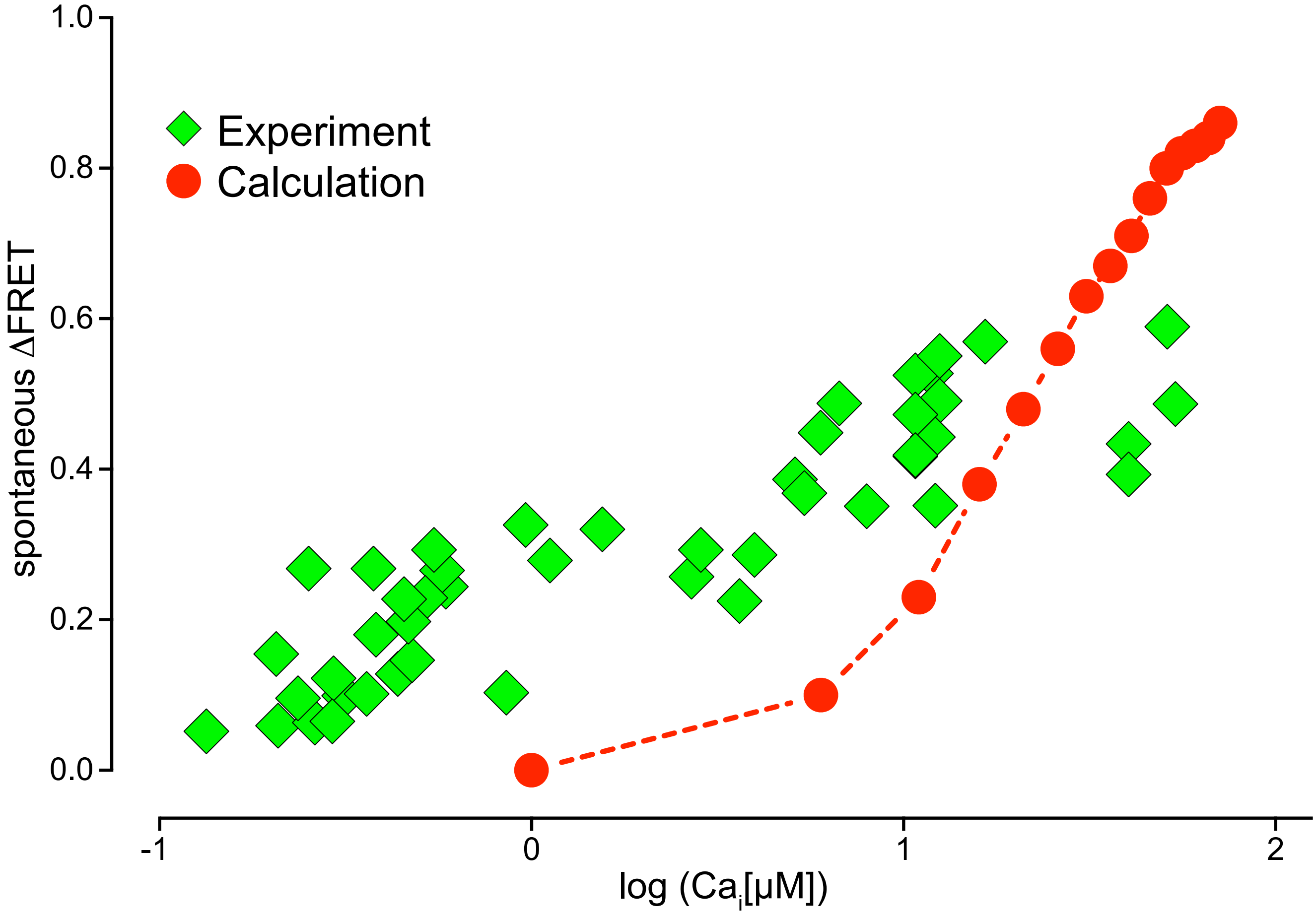

b

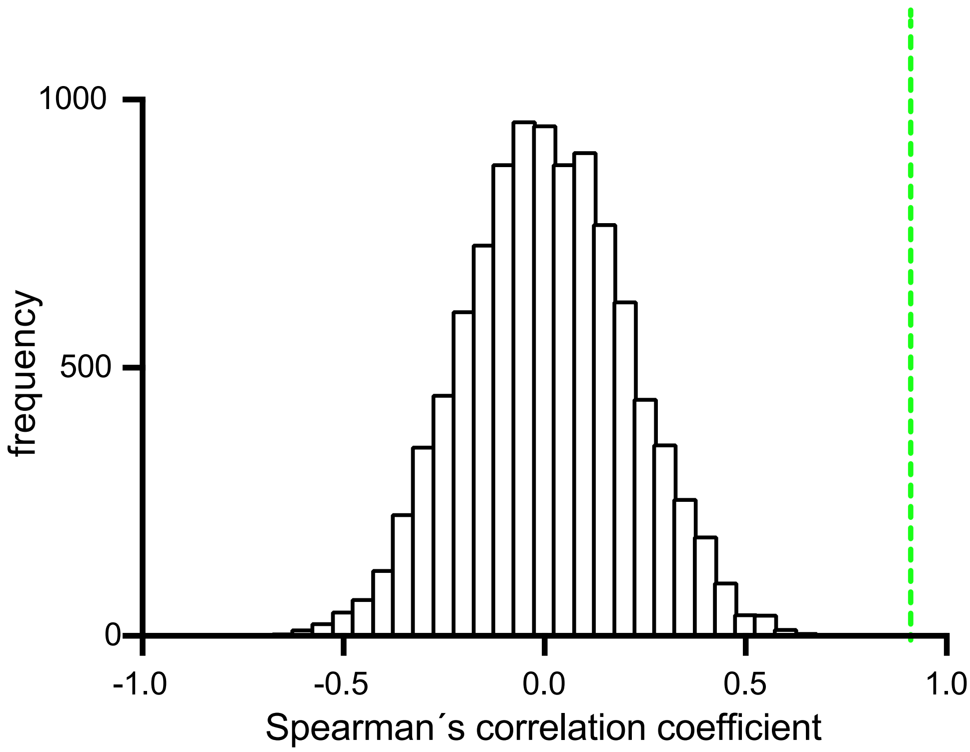

Analysis of spontaneous FRET decay. (a) Amplitude of spontaneous ΔFRET as a function of prolonged elevated Ca_i measured in HEK cells (green diamonds) and derived from the stochastic simulations (red circles).
(b) Distribution of the Spearman correlation coefficients (r) between ΔFRET and Ca_i for 10.000 permutations of the datapairs shown in (a) green diamonds. The dashed green line indicates the correlation level for the measured relationship displayed in (a).

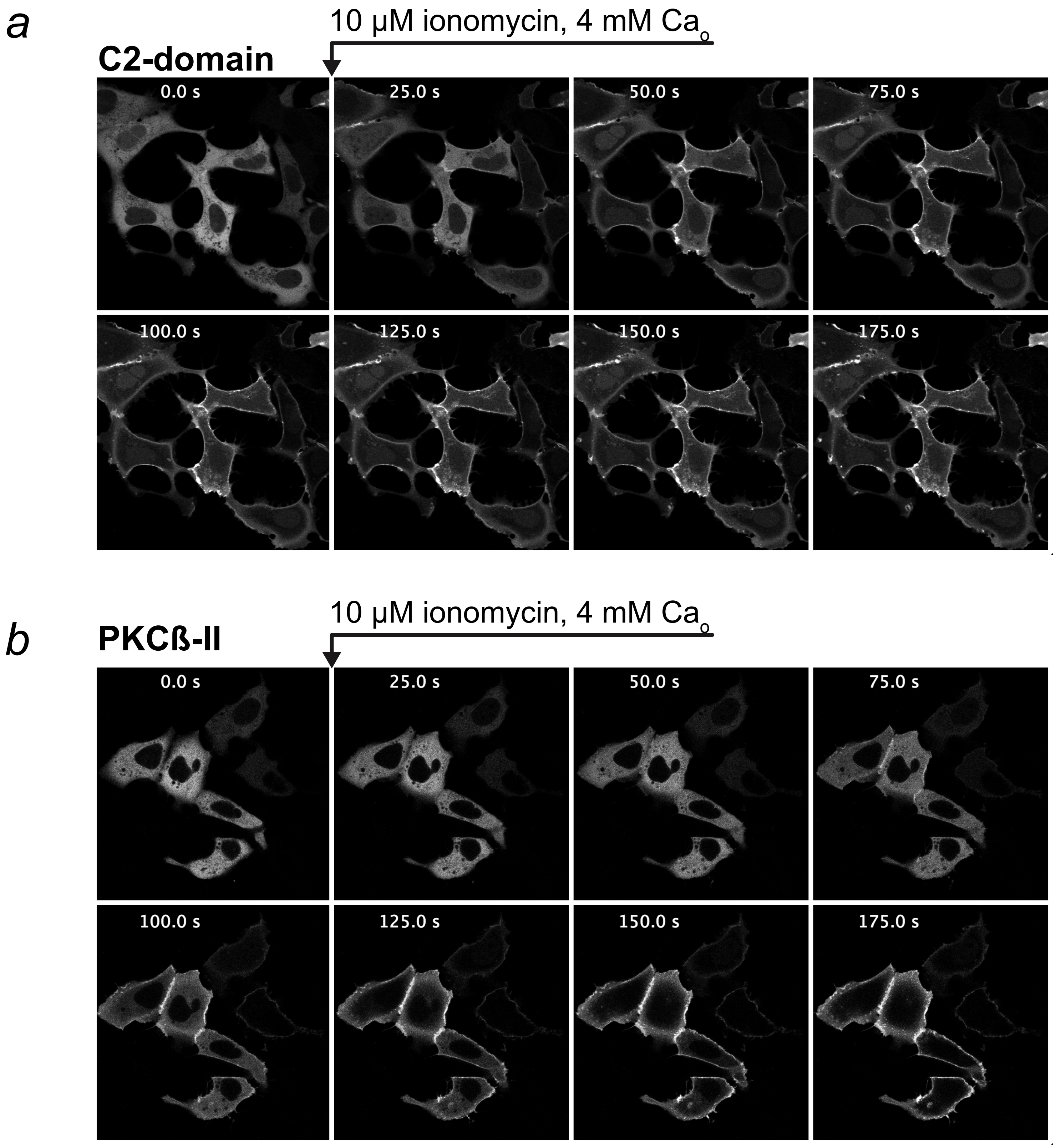

Ca^{2+}-induced C2-domain and PKCß-II translocation to the plasma membrane.
(a) C2-eYFP translocates to the plasma membrane following treatment with 10 µM Ionomycin in 4 mM Ca$_o$
(b) PKCß-II-eYFP targetes to the plasma membrane following treatment with 10 µM Ionomycin in 4 mM Ca$_o$
Start of the treatment is marked by the arrow. Times into the experiment are indicated in the individual images.

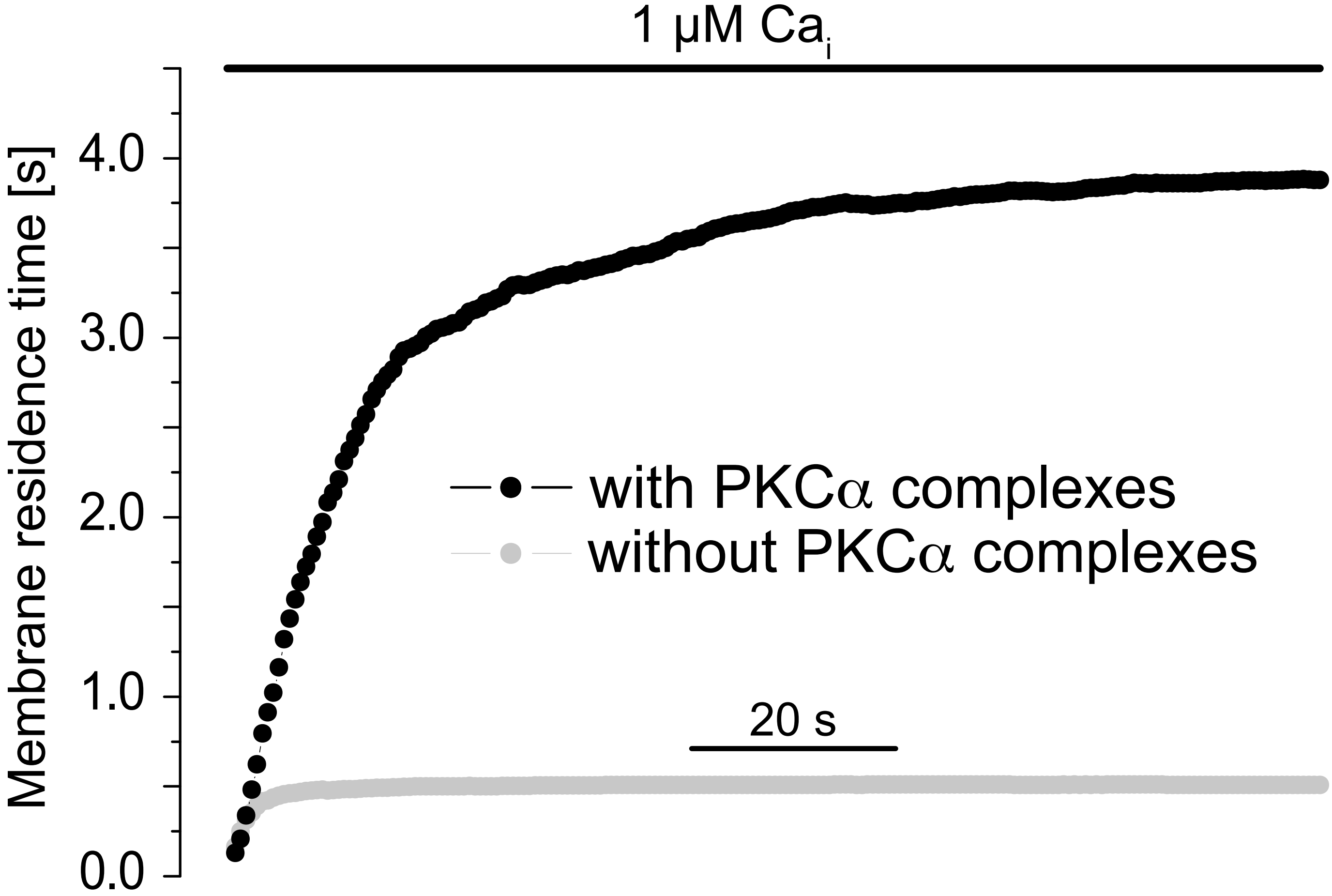

Average membrane residence time of PKCα in simulations using Ca_i=1µM with (black circles) and without (grey circles) intermolecular interactions of PKCα.

Movie 1

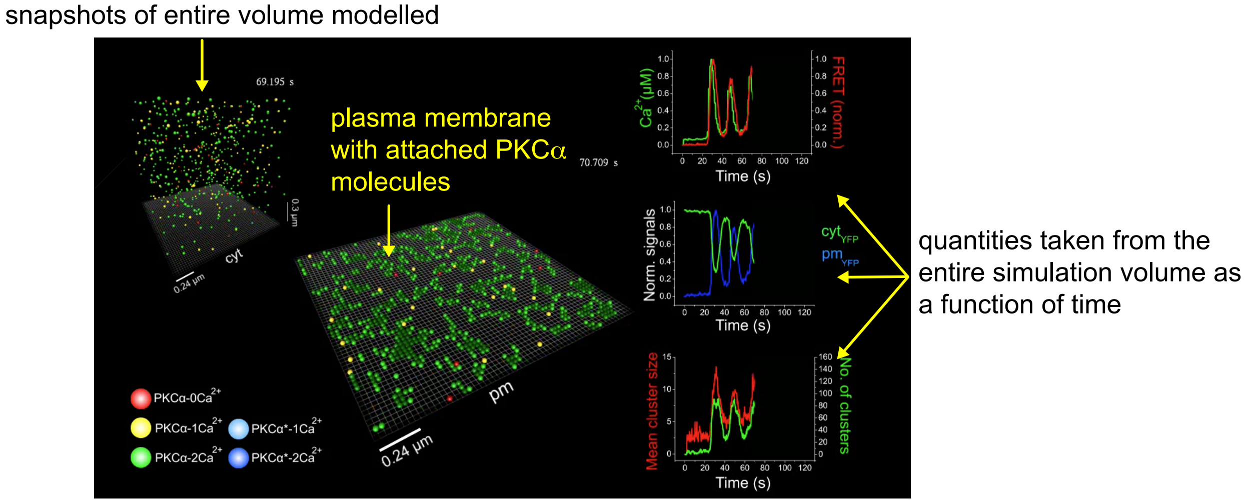

Movie 1: Simulation of PKCα dynamics for the experimental Ca^{2+} signals of Fig. 1d. **Left:** snapshots of the PKCα-particle distribution at indicated time points. **Middle:** simulated plasma membrane area with PKCα-attachement grid and PKCα molecules attached. Color code of the molecules depicted to the lower left. **Right:** Mean cluster size, no of clusters, cytosolic and plasma membrane fluorescence, $[Ca^{2+}]$, and $FRET_{app}$ as a function of time.

Movie 2

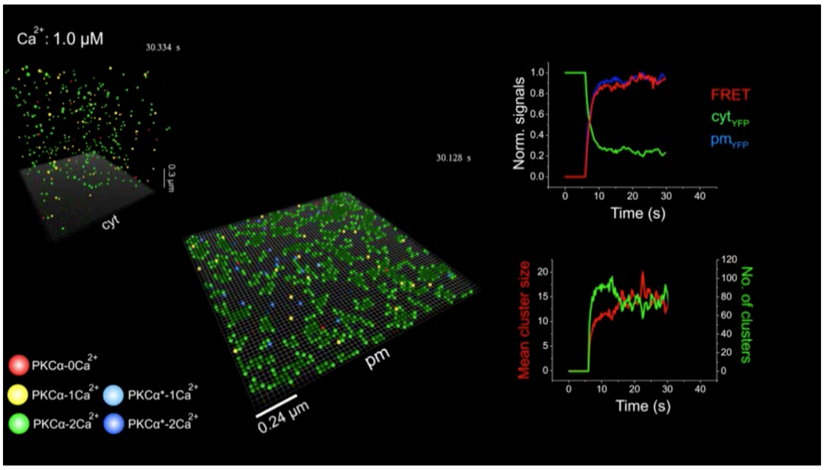

Movie 2: Simulation of PKCα dynamics for a prolonged exposure to low Ca_i (1 μM). **Left:** snapshots of the PKCα-particle distribution at indicated time points. **Middle:** simulated plasma membrane area with PKCα-attachement grid and PKCα molecules attached. Color code of the molecules depicted to the lower left. **Right:** Mean cluster size, no of clusters, cytosolic and plasma membrane fluorescence, $[Ca^{2+}]$, and $FRET_{app}$ as a function of time. Colour code follows the axis legends. Note, that the apparent FRET transient (red) only showed a monotonic increase and subsequent plateau.

Movie 3

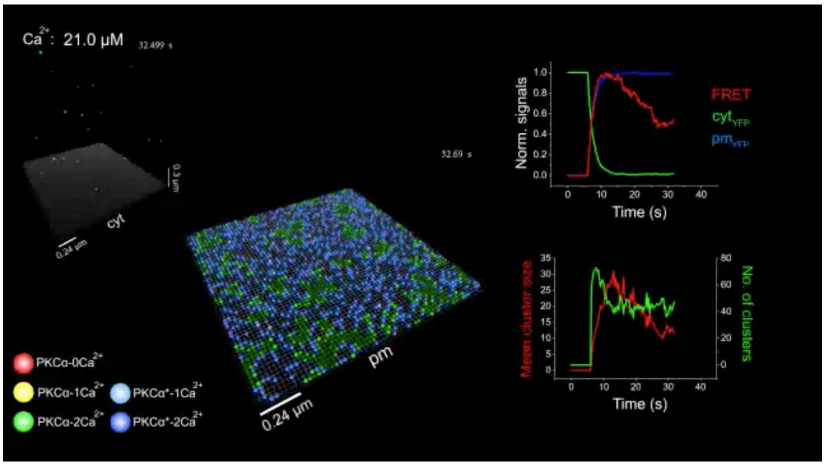

Movie 3: Simulation of PKCα dynamics for a prolonged exposure to high Ca_i (21 μM). **Left:** snapshots of the PKCα-particle distribution at indicated time points. **Middle:** simulated plasma membrane area with PKCα-attachement grid and PKCα molecules attached. Color code of the molecules depicted to the lower left. **Right:** Mean cluster size, no of clusters, cytosolic and plasma membrane fluorescence, $[Ca^{2+}]$, and $FRET_{app}$ as a function of time. Colour code follows the axis legends. Note, that the apparent FRET transient (red) showed a transient peak followed by a lower steady-state plateau level.
